# Investigation of a Rare *CSN1S1*^01^ Allele in Goats Using Allele-Specific PCR and Mathematical Expectation Analysis

**DOI:** 10.3390/ani16050730

**Published:** 2026-02-26

**Authors:** Jingxuan Wang, Yuta Yang, Yuhuan Gao, Ebadu Areb, Xianyong Lan, Haijing Zhu, Chuanying Pan

**Affiliations:** 1 College of Animal Science and Technology, Northwest A&F University, Yangling, Xianyang 712100, China; 2 College of Life Sciences, Yulin University, Yulin 719000, China

**Keywords:** goat *CSN1S1*, AS-PCR, genotyping, mathematical expectation method

## Abstract

The *casein alpha s1* (*CSN1S1*) gene plays an important role in milk protein synthesis and has been associated with milk quality and calcium-related physiological processes in mammals. To date, 19 *CSN1S1* variants have been reported. Among them, the *CSN1S1*^01^ allele has been described as a key “null” variant that may influence goat milk production traits through its effect on *CSN1S1* gene expression. However, the association between this allele and economically important traits in goats remains poorly understood. Therefore, the present study aimed to investigate the *CSN1S1*^01^ allele in goats using economical and rapid approaches, including mathematical expectation (ME) analysis and allele-specific PCR (AS-PCR), and to evaluate its association with economic traits in Chinese goat breeds. A total of 2319 goats from 11 breeds were analyzed. Compared with the “0” allele, the “1” allele showed a higher frequency across all populations and exhibited low genetic diversity. Significant associations were detected between *CSN1S1*^01^ genotypes and body length, third parity, and average kidding number in Laoshan dairy goats (*p* < 0.05). In addition, the low-frequency distribution of the *CSN1S1* “0” allele was further confirmed. Overall, this study provides population-based association evidence supporting the consideration of *CSN1S1*^01^ genetic variation in goat breeding programs.

## Simple Summary

The *casein alpha s1* (*CSN1S1*) gene is a critical milk protein locus in goats, characterized by extensive genetic polymorphism. The *CSN1S1*^01^ allele is a key ‘null’ variant. However, its distribution across diverse breeds and large populations remains unclear. Furthermore, its associations with economic traits have not been fully elucidated. In this study, a cost-effective genotyping approach was employed to screen 2319 goats from 11 Chinese breeds for the *CSN1S1*^01^ allele. Our results show that the “1” allele predominates across all populations, whereas the “0” allele occurs at a low frequency, indicating limited genetic diversity at this locus. Association analysis revealed that *CSN1S1*^01^ genotypes are significantly correlated with body length, third-parity litter size, and average litter size, specifically in Laoshan dairy goats. This study provides important evidence for the association of *CSN1S1*^01^ genetic variation within goat populations.

## 1. Introduction

Casein is a unique phosphoprotein in milk that contains high levels of calcium and phosphorus. In goats (*Capra hircus*), caseins consist of four polypeptide chains: α_s1_, α_s2_, β, and k caseins [1]. The *CSN* genes encoding casein are linked as *CSN1S1-CSN1S2-CSN2-CSN3*, and the gene cluster spans about a 250 kb region on goat chromosome 6 [2,3]. Polymorphisms in these four genes are associated with milk yield and quality traits (fat, protein, and total solids), regulation of milk enzyme activity and edible suitability, body measurement traits, and reproductive traits [4,5,6].

As we know, the α_s1_ -*casein* (*CSN1S1*) is one of four caseins synthesized by the lactating mammary gland. The goat *CSN1S1* gene is generally polymorphic; at least 19 *CSN1S1* protein variants have been identified in goats and used for breed characterization, biodiversity, and phylogenetic research. Detailed information on the identified alleles is summarized in Table 1. Most of the mutations contributing to *CSN1S1* allele differentiation have been characterized. The B1 allele is considered the ancestral form and has given rise to two major lineages: type A (A, A2, A3, 01, 02, I, G, and H) and type B (B2, B3, B4, C, E, F, and L) [7]. Previous studies have suggested that interallelic recombination events between type-A and type-B alleles may have contributed to the formation of the *CSN1S1* N and M alleles [8]. Certain alleles, such as the F allele, are distinguished by specific intronic insertions or deletions [9,10]. Molecular approaches including PCR-SSCP have been widely used to identify alleles such as N and F, and previous studies reported associations with milk yield and composition in Chinese dairy goats [11]. The 02 allele has been described as a null allele containing a large insertion that has not yet been fully characterized. In addition, alleles 01 and 02 are called ‘null’ alleles and cause the deficiency of αs1-casein [12]. An 11 bp indel of this gene was associated with litter size and body measurement traits in goats, suggesting a potential link between *CSN1S1* polymorphisms and multiple traits [6,13]. However, the association between different *CSN1S1* alleles and body measurements and reproductive traits of goats has not been reported. The low-frequency *CSN1S1*^01^ allele of the goat *CSN1S1* gene was mentioned in previous studies, and the mutational origin of this allele is the large deletion of a DNA segment of approximately 8.5 kb, starting from the 181st nucleotide of intron 12 [14]. Although the *CSN1S1*^01^ allele is considered a null allele and has multiple functions such as goat milk allergenicity [15]. Milk obtained from goats carrying the “null” allele may be more suitable for specific nutritional or dietary purposes, specific transformation technology processes, or for “humanized” milk production [16]. Given the diversity of *CSN1S1* gene functions, its influence on growth and reproductive traits requires further exploration. Previous studies have shown that the polymorphism of the *CSN1S1*^01^ allele in goat breeds [17]. Thus, we detected this rare mutation in 11 goat breeds by combining the allele-specific polymerase chain reaction method (AS-PCR) and the mathematical expectation (ME) method [18] and explored the relationship between mutation and its association with growth and reproductive traits.

In this study, we described an economical and rapid method for analyzing the goat *CSN1S1*^01^ allele and found a significant association between different genotypes of the *CSN1S1*^01^ allele and goat growth and reproductive traits. Therefore, this study provides population-based association evidence that may inform future investigations into the role of *CSN1S1*^01^ genetic variation in goat breeding programs.

## 2. Materials and Methods

### 2.1. Samples and Data Collection

In this study, 2319 goats representing 11 breeds were used. Samples of Shaanbei white cashmere goats (SBWC) were collected from Yulin, Shaanxi Province. Guizhou Heima goat (GZHM) and Nubia goat (NG) samples were collected from Guizhou province. Guanzhong (GZ) and Xinong Saanen (SA) dairy goats were collected from Qianyang County in Shaanxi Province. Inner Mongolia white cashmere goats (IMWC) were collected from Ordos City in the Inner Mongolia Autonomous Region. Goats of the same breed were raised and managed under identical conditions, and individuals included in this study were randomly selected. Body measurement traits were recorded following the protocol described by Zhang et al. [24]. Sample collection and storage procedures were performed according to the previously reported method [6].

### 2.2. DNA Isolation and Primer Design

Genomic DNA was extracted from the collected ear tissue samples using the high-salt extraction method [25]. A Nanodrop 2000 spectrometer (Thermo Fisher Scientific, Waltham, MA, USA) was used to determine the DNA purity and quality. Subsequently double-distilled water was added to dilute the extracted DNA to 20 ng/μL and stored at −20 °C. A set of allele-specific primers was designed based on previously published methods to amplify a region spanning part of exon 12 and the adjacent intron 12 of the goat *CSN1S1* gene, which contains the deletion characteristic of the *CSN1S1*^01^ null allele [17]. The primers used were: goat-*CSN1S1*-AS-a (5′-CCCCAGCTGGTAATGTTTTA-3′), goat-*CSN1S1*-AS-b (5′-GGTCCATCAATTCCCTGTGT-3′), and goat-*CSN1S1*-AS-c (5′-TGTATGGATCCCTGATTCCTTC-3′). The amplified fragment is approximately 249 bp for the *CSN1S1*^01^ allele and 281 bp for the reference allele, allowing clear discrimination of homozygous, heterozygous, and reference genotypes. Primer positions relative to the gene structure are illustrated in a schematic diagram. The PCR was performed in a 20 μL reaction mixture containing 2 μL of DNA solution (20 ng) goat genomic DNA (constructed from four different individuals), 10 μL of 2× Eco *Taq* PCR Super mix (Vazyme Biotech Co., Ltd., Nanjing, China; Cat# No. P222-01), 0.6 μL of forward primer, 0.4 μL of reverse primer, and 6.6 μL of double distilled. The protocols for the PCR reaction mixture and amplification conditions used in the present study were based on Yang et al. [26]. A 3.5% agarose gel was used for electrophoresis of the PCR products to identify the indel locus [27].

### 2.3. Statistical Analysis

Previously reported methods were used to examine the Hardy–Weinberg equilibrium (HWE) and population-genetic parameters, such as homozygosity (Ho), effective allele numbers (Ne), and polymorphism information content (PIC) [28]. The association between the goat *CSN1S1*^01^ allele and economic traits was analyzed using *t*-test on SPSS software (version 24.0). A linear model of the relationship between goat genotypes and each growth trait was constructed using Hui’s method [29].

## 3. Results

### 3.1. Identification of Indel Variations and Genotyping

**Figure 1:**
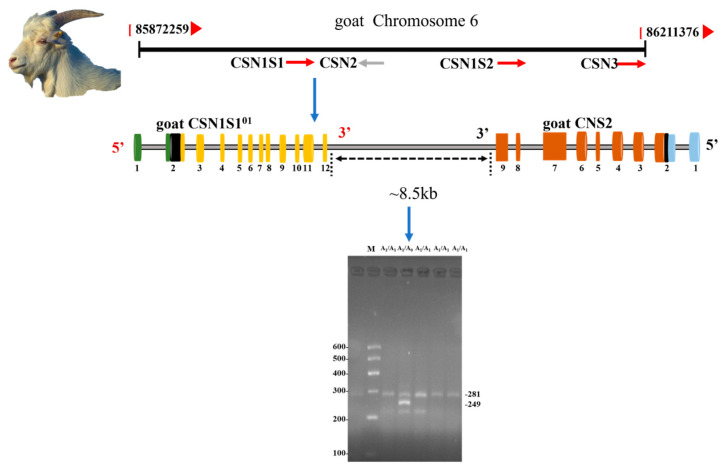
Electrophoresis of AS-PCR products of goat *CSN1S1* gene. Note: Red arrows indicate the transcription orientation of genes, colored boxes represent exonic regions, the blue arrow denotes the deletion locus analyzed in this study, and the dashed line indicates the genomic distance between *CSN1S1* and *CSN2* (~8.5 kb).

The AS-PCR amplification results showed that there were two genotypes in the goat population: the A^1^A^1^ genotype showed one band (281 bp), and the A^1^A^0^ genotypes showed two bands (281 and 249 bp) (Figure 1). However, only the A^1^A^1^ genotype was detected in IMWC, GB, BJ, MT, GW, SBWC, and NG goats (Table 2).

### 3.2. Genetic Parameter Analysis

The frequency of the A_1_ allele ranged between 0.946 and 1.000, and this locus was in Hardy–Weinberg equilibrium in all populations (*p* > 0.05). The genotypic distribution among the 11 goat breeds was determined by calculating the genetic parameters associated with the *CSN1S1*^01^ allele, such as genotype and allele frequencies (Table 2). Compared with the “0” allele, the “1” allele had a higher frequency in the 11 goat breeds. Based on the PIC values, this allele showed low genetic diversity in all breeds.

### 3.3. Association Analysis of Genotypes and Body Measurement Traits

The association between the *CSN1S1*^01^ allele and economic traits in Laoshan dairy goats is shown in Table 3 and Table 4. The *CSN1S1*^01^ allele was highly associated with third parity and average kidding number (*p* < 0.05, Table 3). A doe with A^1^A^0^ genotype produces approximately 0.6 more kids per parity (2.25 vs. 1.61) represent a major economic benefit through more replacement stock. Similarly, the *CSN1S1*^01^ allele was also associated with body length (*p* < 0.05, Table 4). For all other traits (weight, height, chest, cannon), the A^1^A^0^ genotype had numerically higher averages than A^1^A^1^ but statistically non-significant.

## 4. Discussion

In this study, the *CSN1S1*^01^ polymorphism was systematically characterized across multiple goat populations using AS-PCR and ME methods. Only two genotypes (A^1^A^1^ and A^1^A^0^) were detected, and the A^0^ allele was observed at a very low frequency and only in a limited number of breeds. This distribution pattern indicates that *CSN1S1*^01^ is a low-polymorphism locus in goats, which is consistent with previous reports describing the rarity of the A^0^ allele in different geographic and production populations [13,17,30,31].

The investigation of genetic variation in the *CSN1S1* gene and its association with production traits is of considerable interest. Previous studies have demonstrated that polymorphisms in *CSN1S1* are associated with variation in milk casein content as well as milk structure and nutritional properties. To date, ruminants—including cattle, sheep, and goats—remain the primary source of milk for human consumption worldwide, and most studies on *CSN1S1* have therefore focused on these species. In cattle, ten *CSN1S1* protein variants (A, B, C, D, E, F, G, H, I, and J) have been reported, and several alleles have been associated with differences in milk composition traits, such as milk fat percentage and *CSN1S1* content [32,33]. In sheep, seven *CSN1S1* phenotypes (A, B, C, D, E, F, and X) have been identified [34]. Beyond milk composition, *CSN1S1* polymorphisms have also been examined in association studies involving other economically relevant traits in livestock. For instance, *CSN1S1* polymorphisms have been associated with lower yearling weights in cattle [35]. In goats, *CSN1S1* polymorphisms have also been linked to reproductive and growth traits [14,24]. However, the biological basis of these associations remains unclear. Overall, existing evidence indicates that genetic variation at the *CSN1S1* locus is primarily relevant to milk-related traits, while reported associations with non-milk traits should be considered descriptive and population-specific rather than indicative of direct functional effects.

In the present study, we focused specifically on the *CSN1S1*^01^ allele across different goat populations. Methods including ME and AS-PCR were established for reliable identification of the 01 allele, and the reported mutation frequency of this allele in other goat populations is summarized in Table 5. Notably, while the frequency of the *CSN1S1*^01^ allele varies across breeds, it reaches an exceptionally high level (approximately 70–73%) in Norwegian Dairy goats, significantly impacting milk composition and technological properties [36,37]. Genetic parameter analysis revealed that the *CSN1S1* locus adheres to the Hardy–Weinberg equilibrium (HWE) across all studied populations, with PIC values indicating a low genetic diversity. These results suggest a relatively stable allelic distribution and imply that strong directional selection is unlikely to be acting on this locus. However, the limited polymorphism observed necessitates caution when interpreting downstream association analyses, particularly concerning rare genotypes. Association analysis demonstrated that the *CSN1S1*^01^ allele is significantly correlated with the third-parity litter size and body length in Laoshan dairy goats, whereas no significant associations were detected for other morphological traits. Notably, these associations were inconsistent across breeds and traits, highlighting a clear population- and trait-specific pattern. The absence of significant effects on most growth-related parameters suggests that *CSN1S1* polymorphisms do not exert a broad influence on overall body conformation. Reproductive performance and body measurements are complex quantitative traits governed by polygenic inheritance and environmental-management interactions. The parity-specific associations observed here further reflect the complexity of reproductive traits, potentially influenced by sample structure, breeding history, or management practices. Consequently, the identified associations should be interpreted as localized statistical correlations rather than evidence of a universal or direct genetic effect.

From a methodological perspective, this study establishes a practical framework for the detection and population-level screening of the *CSN1S1* allele in goats. The integrated application of AS-PCR and ME methods ensures reliable genotype differentiation across diverse breeds, facilitating future investigations into *CSN1S1* polymorphisms within various genetic backgrounds. However, several limitations must be acknowledged. First, the low frequency of the A^0^ allele constrained the statistical power of our association analyses, particularly in breeds where only the A^1^A^1^ genotype was present. Second, this study focused on a single locus without integrating functional or expression data. Finally, environmental and management factors, which can influence reproductive performance and growth traits [40], should be considered in future investigations as their potential effects were not explicitly modeled here. These constraints underscore the necessity for further validation in independent cohorts and integrated omics research to fully elucidate the biological significance of *CSN1S1* variations.

In summary, this study systematically characterized the genetic variations in *CSN1S1* across Chinese goat populations and elucidated population-specific associations with key growth and reproductive traits. While these findings are preliminary and descriptive in nature, they expand the current understanding of *CSN1S1* polymorphisms and provide a valuable foundation for subsequent functional validation.

## 5. Conclusions

AS-PCR and mathematical expectation (ME) methods can be used to rapidly and efficiently detect goat *CSN1S1*^01^ allelic mutations. The low-frequency characteristics of the *CSN1S1*^01^ allele are universal in the goat breeds. The *CSN1S1*^01^ allele shows population- and breed-specific associations with selected growth and reproductive traits in Laoshan dairy goats.

## Figures and Tables

**Table 1:** Details on the goat *CSN1S1* alleles.

Alleles	Variations	References
A	Glu (77) → Gln	[19]
A2	uncharacterized	NA
A3	uncharacterized	NA
B1	Reference 199 residues	[20]
B2	Leu (16) → pro	[21]
B3	Leu (16) → pro, Arg (100) → Lys	[21]
B4	Leu (16) → pro, Arg (100) → Lys, Thr (195) → Ala	[21]
C	Leu (16) → Pro; Arg (100) → Lys; Thr (195) → Ala; His (8) → Ile	[19]
D	Leu (16) → Pro, deletion 59 to 69	[19]
E	a 457 bp insertion within exon 19 (last untranslated exon)	[22]
F	presence of short insertions of 11 bp (CGTAATGTTTC) (nucleotides 9972–9982) and 3 bp (AAT) (nucleotides 10,639–1042) inside the 9th intron, and by the deletion of 7 bp (TTATCTA) at the 14th intron between the 14,647 and 14,648 nucleotides	[9]
G	Glu (77) → Gln, deletion 14 to 26	[21]
H	Glu (77) → Gln; Arg (1) → Lys	[9]
I	unknown	[23]
L	Leu (16) → Pro; Arg (90) → His	[23]
M	Ser (66) → Leu a single nucleotide transition C → T (nucleotide 23/exon 9)	[23]
N	the deletion of cytosine at the 23rd nucleotide of the 9th exon, without the insertion of 11 and 3 bp in the subsequent intron	[8]
01	the deletion of a DNA segment of about 8.5kb starting from the 181st nucleotide of the intron 12, and including the last 7 exons of the gene	[9]
02	A large insertion, so far uncharacterized	NA

“NA” indicates: Not Available.

**Table 2:** The population genetic metrics of the *CSN1S1* locus for different populations.

Types	Breeds	A^1^A^1^ Genotype	A^1^A^0^ Genotype	Total	A^1^/A^0^ Alleles	Ho	He	Ne	PIC	X^2^ Value
Cashmere type	IMWC	689	14	703	0.985/0.015	0.969	0.031	1.032	0.03	0.11
	SBWC	1031	0	1031	1.000/0	1	0	1	0	0
Meat type	GZHM	200	1	201	0.995/0.005	0.995	0.005	1.005	0.005	0.001
	NG	69	0	69	1.000/0	1	0	1	0	0
	GZBG	21	0	21	1.000/0	1	0	1	0	0
	BJ	25	0	25	1.000/0	1	0	1	0	0
	MT	22	0	22	1.000/0	1	0	1	0	0
	GW	31	0	31	1.000/0	1	0	1	0	0
Dairy type	Sa	66	8	74	0.946/0.054	0.898	0.102	1.114	0.097	0.24
	LS	76	4	80	0.975/0.025	0.951	0.049	1.051	0.048	0.05
	GZ	60	2	62	0.984/0.016	0.968	0.032	1.033	0.031	0.02

Note: Sa = Saanen dairy goat, LS = Laoshan dairy goat, GZ = Guanzhong dairy goat, IMWC = Inner Mongolia white cashmere goat, GZBG = Guanzhong black goat, BJ = Banjiao goat, MT = matou goat; GW = Guizhou white goat; SBWC = Shaanbei White Cashmere Goat; GZHM = Guizhou heima goat; NG = Nubia goat.

**Table 3:** Association between different genotypes of the *CSN1S1* gene with litter size (means ± SE) in Laoshan dairy goats.

Fixed Effects	A^1^A^1^ Genotype (*n* = 76)	A^1^A^0^ Genotype (*n* = 4)	*p*-Value
The third parity	1.67 ± 0.10 ^a^	2.50 ± 0.29 ^b^	0.02
Average	1.61 ± 0.08 ^a^	2.25 ± 0.43 ^b^	0.05

Note: Different lowercase letters (a, b) indicate significant differences at *p* < 0.05. “The third parity” refers to the number of kids born at the third kidding. “Average” refers to the mean number of kids per kidding.

**Table 4:** Association between different genotypes of *CSN1S1* gene and goat economic traits (Mean ± SE) in Laoshan dairy goat.

Traits	A^1^A^1^ Genotype (*n* = 76)	A^1^A^0^ Genotype (*n* = 4)	*p*-Values
body weight (kg)	47.30 ± 1.09	51.00 ± 5.00	0.490
body height (cm)	76.05 ± 0.52	80.50 ± 0.50	0.119
body length (cm)	78.29 ± 0.52 ^a^	85.50 ± 1.50 ^b^	0.014
chest circumference(cm)	79.34 ± 0.52	83.00 ± 0.53	0.215
cannon circumference (cm)	9.63 ± 0.13	10.00 ± 0.12	0.601

Note: Different lowercase letters (a, b) indicate significant differences at *p* < 0.05.

**Table 5:** The allele frequency of the *CSN1S1* locus for the reported populations.

Breeds	Total	A^1^/A^0^ Alleles	References
GSB (White shorthaired)	123 333	0.976/0.024 0.981/0.019	[12] [12]
BSH (Brown shorthaired)	45 165	0.989/0.011 0.985/0.015	[12] [12]
Sarda goats	935	0.999/0.001	[31]
Frisa	70	0.893/0.107	[30]
Orobica	66	0.977/0.023	[30]
Verzasca	67	0.993/0.007	[30]
Camosciata	88	0.9890/0.011	[30]
Neapolitan goats	285	1.000/0	[7]
Alpine breeds	83	0.990/0.010	[38]
Kilis	60	1.000/0	[39]
Sanliurfa	66	1.000/0	[39]
Siirt	55	1.000/0	[39]
Norwegian dairy goat	575	0.263/0.737	[36,37]

## Data Availability

All data analyzed during this study are available from the corresponding author upon reasonable request.

## Abbreviations

Sa: Saanen dairy goat; LS: Laoshan dairy goat; GZ: Guanzhong dairy goat; IMWC: Inner Mongolia white cashmere goat; GZBG: Guanzhong black goat; BJ: Banjiao goat; MT: matou goat; GW: Guizhou white goat; SBWC: Shaanbei White Cashmere Goat; GZHM: Guizhou Heima goat; AS-PCR: allele-specific polymerase chain reaction; ME: mathematical expectation.
